# Ecotoxicological Evaluation of Organic Sunscreens Used Worldwide, Alone and in Mixture, on Terrestrial Plants

**DOI:** 10.1002/tox.24571

**Published:** 2025-10-07

**Authors:** Diego Espirito Santo, Edson Araújo de Almeida, Elisângela Dusman, A. C. Downs, Regiane da Silva Gonzalez, Osvaldo Valarini Junior, Ana Paula Peron

**Affiliations:** ^1^ Postgraduate Program in Environmental Engineering Federal Technological University of Paraná Paraná Brazil; ^2^ Postgraduate Program in Chemistry Maringá State University Paraná Brazil; ^3^ Haereticus Environmental Laboratory Gladstone Virginia USA; ^4^ Postgraduate Program in Food Technology Federal Technological University of Paraná Paraná Brazil; ^5^ Postgraduate Program in Technological Innovations Federal Technological University of Paraná Paraná Brazil

**Keywords:** binary mixtures, cultivated plants, oxidative stress, toxicity, ultraviolet filters

## Abstract

The environmental hazards of sunscreens are discussed worldwide. However, there are few ecotoxicological studies on these compounds alone for edaphic organisms, and none for their mixtures. Avobenzone (1 and 10 ng/L), octocrylene (10 and 100 μg/L), and oxybenzone (2 and 20 μg/L), alone and in binary combinations (between the lowest and the highest concentrations), were evaluated for phytotoxicity to the roots of rustic varieties of *Daucus carota* and *Solanum lycopersicum*, and for cytotoxicity and genotoxicity in the roots of *Allium cepa* bulbs. In contrast to the higher concentrations, the lower concentrations of sunscreens, despite the increase in superoxide and hydroxyl radicals in the cells, did not cause changes in the length of the rootlets, since they did not affect cell elongation. In mixture, the lower concentrations caused a synergistic interaction in the roots, while the higher did not exceed the toxicity of the filters alone. In bulbs, the filters alone and in mixtures caused inhibition of cell division and mitotic spindle alterations in the meristems, mainly due to the accumulation of H_2_O_2_ in the cells, and the mixtures triggered a synergistic interaction in the roots. The mixtures were highly hazardous, especially avobenzone‐oxybenzone, the most absorbed by the roots and with the greatest phytotoxic and cytogenotoxic potential; however, of low environmental stability. Environmentally stable mixtures with octocrylene were the least absorbed but were highly harmful, inducing phytotoxicity and cytogenotoxicity in the plants. Therefore, the use of sewage sludge and wastewater on crop soils poses a risk to agricultural productivity and the environment.

## 1 Introduction

Organic sunscreens have become indispensable in the prevention of skin cancer and skin aging. These compounds are present in several sunscreen lotions cosmetics, and pharmaceutical products [1]. Despite their importance, they are classified as micropollutants since they are not included in environmental regulations, as information on their harmfulness to different ecosystems is still incipient [1, 2].

Among the most widely used sunscreens in the industry are avobenzone (AVB), effective against ultraviolet radiation between 350 and 365 nm, octocrylene (OCT), and oxybenzone (OXB), effective against ultraviolet radiation between 280 and 360 nm. These compounds are organic molecules with conjugated aromatic structures, of molecular weights 310.39, 361.49, and 228.5 g/mol, and log Kow values of 4.51, 6.88, and 3.79, respectively, and are therefore highly lipophilic and bioaccumulative [3, 4, 5]. OCT is the most commonly found filter in water and soil because it has dual function in commercialized products, to effectively protect against photodegradation and to promote the stability and functionality of other filters such as AVB, OXB, benzophenone‐4, and octinoxate [6, 7, 8]. In the environment, AVB photodegrades rapidly, but this compound is considered pseudo‐persistent because it is recurrently released into different environmental matrices [6]. OCT and OXB are resistant to photodegradation and have low biodegradability in water and soil [6, 9].

In aquatic environments, the presence of AVB, OCT, and OXB, in concentrations ranging from ng to μg/L, is mainly due to the discharge of domestic and industrial effluents into water resources due to inefficient water treatment for micropollutants [2, 10]. In the soil, they are mainly found by incorporating domestic sewage sludge into agricultural soils and using wastewater for irrigation [7, 11].

It should be noted that global sewage sludge production is estimated at 45 million megagrams (Mg) of dry matter per year. In European Union countries alone, production has increased by 1.5 million Mg over the past 10 years, and more than 50% of this amount is used in agriculture [12, 13]. In Brazil, an average of 220 000 tons of sludge is produced annually, and 90% is used on farmland, mainly for soybeans and corn, as well as in extensive vegetable gardens [14, 15]. Furthermore, more than 10% of the world's population already consumes agricultural products irrigated with wastewater, but most countries do not have rules for effluent reuse [16]. In Brazil, it is estimated that the wastewater generated in the country under conditions of reuse is equivalent to 9% of the total water demand for agriculture, which is 52% [17]. In different countries, AVB, OCT, and OXB have been found in wastewater and sewage sludge at concentrations up to 2 μg/L and 212.9 μg/L, 201.9 μg/L and 999.1 μg/L, 500 μg/L and 1000 μg/L, respectively [5, 7, 8, 18].

In aquatic organisms, AVB, OCT, and OXB alone have caused adverse effects in protozoa, microcrustaceans, fish, mollusks corals, and algae, such as metabolic changes, reduction in fertility rate, mortality, and bioaccumulation [10, 19, 20, 21, 22]. In terrestrial organisms, the few ecotoxicological studies carried out were on soil organisms and have shown that AVB, OCT, and OXB individually have the potential to induce physiological, cellular, and biochemical changes in plants and earthworms [2, 5, 7, 8, 18, 23].

However, products formulated with sunscreens active‐ingredients often contain a combination of two or more sunscreen filters, and in different environmental matrices they are found in mixture [10]. Although it is important to understand how sunscreens act individually on different species, it is prudent to evaluate them in mixture to understand the interactions—additive, synergistic, and/or antagonistic—between them in bioassays representative of different trophic levels [9, 10]. The studies reported in the literature on the combined ecotoxicity of organic sunscreens mostly involve aquatic organisms, in which filters were tested in binary and/or tertiary combinations [24, 25, 26]. To date, there are no ecotoxicological evaluation studies of organic sunscreens in mixtures in soil organisms, becoming an imminent environmental issue to be understood, since soils worldwide are repeatedly contaminated with these compounds.

*Daucus carota* L. (carrot) and *Solanum lycopersicum* L. (tomato) are standard plant species in ecotoxicological studies of environmental contaminants, such as emerging pollutants, where their varieties rustics and no rustics are evaluated for physiological and biochemical parameters [8, 18, 27, 28, 29] and recommended by the United States Environmental Protection Agency (USEPA) (1996) [30] and the Organization for Economic Cooperation and Development (OECD) (2006) [31]. *Allium cepa* L. (onion) roots are a robust bioassay that has been used worldwide for more than five decades to assess the systemic and cellular toxicity of environmental contaminants, such as micropollutants, and for environmental monitoring [5, 32, 33, 34]. Among its important advantages as a biological model is the high similarity of its physiological, cytological, and biochemical results with other plants, mammals, and in vitro tests [7, 18, 32, 33, 34, 35, 36, 37, 38, 39].

This study evaluated the systemic and cellular toxicity induced by AVB, OCT, and OXB, individually and in binary mixtures, in *D. carota*, *S. lycopersicum*, and *A. cepa* plants at environmentally relevant concentrations using multiple biomarkers. This study is pioneering in the ecotoxicological evaluation of sunscreen mixtures in plants. This study also provides additional and important ecotoxicological information on the isolated effects of these compounds in these organisms. These results contribute to the understanding of the environmental impacts caused by sunscreen mixtures and will assist in the construction of regulations that limit the release of these compounds into the environment.

## 2 Material and Methods

### 2.1 Obtaining the Sunscreens, Preparing, and Defining the Concentrations for the Study

AVB (butylmethoxydibenzoylmethane, CAS 70356‐09‐1), OCT (2‐ethylhexyl‐2‐cyano‐3,3‐diphenylacrylate, CAS 6197‐30‐4), and OXB (2‐hydroxy‐4‐methoxybenzophenone or benzophenone‐3, CAS 131‐57‐7) were purchased analytical grade from Sigma‐Aldrich, as were the other reagents used in this study.

The concentrations of sunscreens for study were prepared in an aqueous medium, in which Tween 80 (in the same mass concentration as the filters) was used as a solubilizer.

The concentrations for carrying out in vivo and *ex situ* tests in plants were defined based on information on the concentrations of AVB, OCT, and OXB in sewage sludge and wastewater, previously mentioned. In order to approach a more realistic scenario regarding soil contamination with sunscreens, the concentrations defined for study were: AVB 1 and 10 ng/L, OCT 10 and 100 μg/L, and OXB 2 and 20 μg/L.

### 2.2 Stability Analysis of AVB, OCT, and OXB in Aqueous Media for Seven Days

AVB, OCT, and OXB stock solutions, kept in the dark and at room temperature, were individually evaluated for stability in aqueous media over seven days (Equation 1). Analyses were performed in UV–Vis spectrophotometer at 350, 280, and 275 nm, respectively. The results were expressed as percentages.

(1) $$S\left(\%\right)=\frac{A_{s}}{A_{0}}\times 100$$

where *S* is the stability, *A*_s_ is the absorbance of the sample, and *A*_0_ is the initial absorbance on day 0.

### 2.3 Evaluation of Phytotoxicity in *D. carota* and *S. lycopersicum* Seeds

Germination tests were performed according to OECD (2006) [31] with slight adaptations. Seeds of *D. carota* (rustic variety, elongated orange), brand Isla, and *S. lycopersicum* (rustic variety, round), brand Feltrin, free of agrochemicals were used. According to the labels, the seed lots had a germination rate of over 95% and a purity of between 99.8% and 100%. Seeds from the same batch were used throughout the experiment.

The carrot and tomato varieties used in this study are classified as rustic because they are found in natural populations and show greater adaptation to environmental adversity than non‐rustic varieties.

For phytotoxicity analysis, seeds of each species were distributed in previously sterilized Petri dishes lined with filter paper. Twenty seeds were used per plate and each treatment was analyzed in quintuplicate. In each plate, the filter paper was irrigated with the treatment solution (1.5 mL), taking care not to soak it. The plates were then wrapped in plastic to prevent drying out and placed in a Biochemical Oxygen Demand (BOD) incubator at 25°C, where they remained in the dark. Distilled water was used as control.

A seed was considered to have germinated when the radicle emerged. The percentage of germination was calculated according to (Equation 2).

(2) $$G\left(\%\right)=\frac{\mathrm{GS}}{\mathrm{TS}}\times 100$$

where *G* is the germination, GS is the number of seeds germinated, and TS is the total number of seeds used.

After seven days, the seed roots were measured with a digital caliper, and the Relative Growth Index was calculated (Equation 3).

(3) $$\mathrm{RGI}=\frac{\mathrm{RLI}}{\mathrm{RLC}}$$

where RGI is the Relative Growth Index, RLI the average length of the roots exposed to the treatment, and RLC the average length of the control roots.

According to the protocol of Biruk et al. (2017) [40], RGI values between 0.8 and 1.2 (0.8 ≤ RGI ≤ 1.2) indicate that the treatments did not affect root elongation, while values below 0.8 (0.1 < RGI < 0.8) indicate inhibition of root growth, and values above 1.2 (RGI > 1.2) indicate stimulation of root growth.

The Germination Index was calculated according to (Equation 4). According to Mañas and Heras (2018) [41], germination indices less than or equal to 50% (GI ≤ 50%) indicate high risk for the plant, values between 50% and 80% (50% < GI < 80%) indicate moderate risk and values greater than or equal to 80% (GI ≥ 80%) indicate low risk.

(4) $$\mathrm{GI}\left(\%\right)=\frac{\mathrm{RLI}\times \mathrm{GSI}}{\mathrm{RLC}\times \mathrm{GSC}}\times 100$$

where GI is the Germination Index, RLI the average length of roots exposed to treatment, RLC the average length of roots in the control, GCI the number of seeds germinated under exposure to treatment, and GSC the number of seeds germinated in the control.

### 2.4 Evaluation of Cytotoxicity and Genotoxicity in *A. cepa* Roots

The tests with *A. cepa* (variety baia periforme) followed the protocol of Fiskesjö (1985) [42] with adaptations by Filipi et al. (2023) [15]. The bulbs were purchased from an organic garden. The dehydrated cataphylls and dried roots were removed, and the bulbs were washed under running water. The bulbs were then placed in beakers containing the treatment solutions where the root growth zone was immersed in the solution. The onions were then placed in a BOD incubator in the dark for five days. Treatment solutions were prepared and changed daily. Distilled water was used as a control, and five bulbs were used for each treatment.

At the end of the incubation period, roots from each bulb were collected and fixed in Carnoy 3:1 for 24 h. After this time, slides of the meristematic regions of the roots were mounted and analyzed under an optical microscope with a 40× objective lens.

To assess the cytotoxicity, 2000 cells from each bulb were analyzed, giving a total of 10 000 cells per treatment, and the Mitotic Index was calculated (Equation 5). To assess genotoxicity, 200 cells from each bulb were analyzed, giving a total of 2000 cells per treatment, and the Cell Alteration Index (CAI) was calculated (Equation 6). The cellular alterations considered were multipolar spindles and polyploidy, micronuclei, chromosomal abnormalities in prophase, metaphase, anaphase and telophase, viscosity, chromosomal bridges in anaphase and telophase, and chromosomal breaks.

(5) $$\mathrm{MI}=\frac{\mathrm{DC}}{\mathrm{TC}}\times 100$$

(6) $$\mathrm{CAI}=\frac{\mathrm{AC}}{\mathrm{TC}}\times 100$$

where MI is the Mitotic Index, DC the total number of dividing cells, TC the total number of cells analyzed, CAI the Cell Alteration Index, and AC the total number of cells with cell alterations.

### 2.5 Enzyme Analysis in Roots of *D. carota* , *S. lycopersicum* , and *A. cepa*

For enzymatic analyses, 50 mg of root from each plate or bulb obtained in 2.3 and 2.4 were macerated in 1 mL HCl (38%) and 2 mL diethylenetriaminepentaacetic acid (5 mM) and centrifuged at 4000 rpm for 15 min. The supernatants (enzyme extracts) were analyzed in a UV–Vis spectrophotometer for modulation of the enzymes catalase (CAT), ascorbate peroxidase (APX), guaiacol peroxidase (GPOX), and superoxide dismutase (SOD).

The CAT analysis was based on Kraus et al. (1995) [43] with adaptations from Azevedo et al. (2002) [44]. 2.5 mL of sodium phosphate buffer (pH 7.8) was added to 100 μL mg of extract. For reading at 240 nm, 1 mL of hydrogen peroxide (H_2_O_2_) (1 mM) was added to the samples. The extinction coefficient used for calculation was 2.8 M/cm, and the results were expressed in μmol/min/μg protein (Equation 7).

(7) $$U=\frac{\left[\frac{\left(\frac{A}{t}\right)}{E}\right]\times V_{e}\times \mathrm{DF}}{P}$$

where *U* is the enzyme unit, *A* is the measured absorbance, *t* is the analysis time (1 min), *E* is the extinction coefficient, Ve is the enzyme volume, DF is the dilution factor, and *P* is the protein, obtained from the mass of roots used.

APX analysis was performed according to Zhu et al. (2022) [45]. 2.5 mL sodium phosphate buffer, 500 μL ascorbic acid (0.25 mM), and 1 mL H_2_O_2_ (1 mM) were added to the extracts. Readings were taken at 290 nm. The extinction coefficient was 2.8 M/cm, and the results were expressed in μmol/min/μg protein (Equation 7).

The GPOX analysis was carried out according to Matsuno and Uritani (1972) [46]. In 300 μL of extract, 2.5 mL of sodium phosphate buffer, 250 μL of 0.1 M citric acid, and 250 μL of 0.5% guaiacol were added. Then 250 μL of 1 mM H_2_O_2_ was added to the mixtures, which were vortexed and placed in an oven at 30°C for 15 min. The samples were then placed in an ice bath for 10 min, and 250 μL of 2% sodium metabisulphite was added. The samples were read at 450 nm. The extinction coefficient was 26.6 M/cm, and the results were expressed in μmol/min/μg protein (Equation 7).

SOD analysis was performed according to Sun et al. (1988) [47]. Samples were prepared in duplicate, with half of the extract aliquots kept under 80 W fluorescent light for 20 min and the other half kept in the dark. Aliquots of 200 μL of the samples were supplemented with 0.8 mL sodium phosphate buffer, 500 μL 0.1 mM ethylenediaminetetraacetic acid (EDTA), 500 μL methionine, 500 μL nitro blue tetrazolium (NBT), and 200 μL riboflavin. Samples were read at 560 nm, and results were expressed as *U* (Equation 8).

(8) $$\mathrm{SOD}=\frac{\left(\frac{B_{l}-s_{l}}{B_{l}}\right)-\left(\frac{B_{e}-s_{e}}{B_{e}}\right)}{50}$$

where *B*_l_ is the absorbance of the blank kept in the light, prepared without the enzyme extract, *s*_l_ is the absorbance of the sample kept in the light, *B*_e_ is the absorbance of the blank kept in the dark, and *s*_e_ is the absorbance of the sample kept in the dark. The quotient 50 represents the amount of enzyme required to inhibit 50% of the photoreduction of NBT.

### 2.6 Analysis of Antioxidant Activity (DPPH), phenolic Content (Folin–Ciocalteu), and Lipid Peroxidation (TBARs) in Root Meristems, and Absorption of Treatments by *A. cepa* Roots

For the DPPH, FC, and TBARs assay, 50 mg of root meristems from each bulb of each treatment was used, as obtained in 2.4. The meristems were macerated in distilled water for 15 min and centrifuged at 4000 rpm. The supernatant (homogenate) was collected for analysis. The same procedure was performed for the absorption test, but with whole roots. Analyses were carried out in a UV–Vis spectrophotometer.

#### 2.6.1 DPPH Assay

The antioxidant activity of the roots was assessed according to Unalan et al. (2021) [48], where 250 μL of 0.00316% (m/V) DPPH solution was added to 50 μL of the homogenate. The mixtures were kept in the dark for 30 min. After this time, the mixtures were spectrophotometrically evaluated at 515 nm. (Equation 9) was used to determine the antioxidant activity.

(9) $$A\left(\%\right)=\frac{A_{c}-A_{s}}{A_{c}}\times 100$$

where AA% is the antioxidant activity, A_c_ is the absorbance of the DPPH solution without the sample, and A_s_ is the absorbance of the sample with DPPH.

#### 2.6.2 Folin–Ciocalteu (FC) Assay

FC was analyzed according to the protocol of Carmona‐Hernandez et al. (2021) [49]. In 50 μL homogenate was added 100 μL FC (2 mM methanolic solution of phosphotungstic and phosphomolybdic acids), 50 μL ethanol, and 50 μL distilled water. The mixtures were kept in the dark for 10 min. Then, 50 μL of saturated sodium bicarbonate solution was added to the mixtures, which were left in the dark for more 50 min. They were then analyzed spectrophotometrically at 745 nm.

#### 2.6.3 TBARs Assay

Lipid peroxidation in the roots was analyzed according to Papastergiadis et al. (2012) [50]. In 50 μL of homogenate, 250 μL of TBARs solution (46 mM) was added. The mixture was kept water bath at 90°C for 35 min. After cooling, the samples were analyzed spectrophotometrically at 532 nm.

#### 2.6.4 Absorption of Treatments by *A. cepa* Roots

For this evaluation, the maximum absorbance of the filters present in the root homogenates was determined at 350 nm for AVB, 275 nm for OXB, 280 nm for OCT, 305 nm for AVB‐OCT and OXB‐OCT, and 290 nm for AVB‐OXB. An analytical curve was constructed for the pure sunscreens and their mixtures at different concentrations ranging from 200 to 2.00 μg/mL, and an R^2^ of 0.99 was obtained.

### 2.7 Statistical Analysis

Phytotoxicity, cytotoxicity, genotoxicity, DPPH, FC, TBARs, and antioxidant enzyme data were subjected to Kruskal‐Wallis analysis of variance followed by Dunn's test (*p* ≤ 0.05) using RStudio software.

## 3 Results and Discussion

### 3.1 Stability in Aqueous Media

**FIGURE 1:**
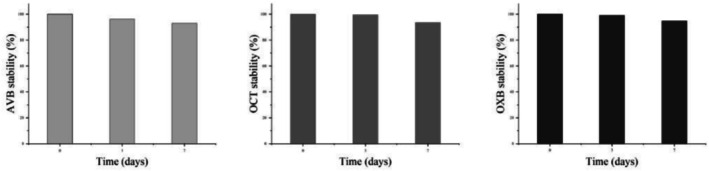
Stability of avobenzone (AVB), octocrylene (OCT), and oxybenzone (OXB) in aqueous media for seven days in the absence of light.

The stability of AVB, OCT, and OXB alone over 7 days was determined (Figure 1). The results are presented in percentages, and the concentrations were equal to 100% at time zero. The three sunscreens remained stable throughout the analysis period.

### 3.2 Toxicity and Oxidative Stress in Seeds of *D. carota* and *S. lycopersicum*

Table 1 shows that AVB, OCT, and OXB, alone and in binary combinations, did not cause any damage to the germination of carrot and tomato seeds at the two concentrations evaluated. The lowest concentrations of AVB, OCT, and OXB alone in carrot and tomato did not cause a reduction in root length. However, their roots were more fragile than the control's, breaking when touched lightly. At higher concentrations, the three filters alone caused a significant reduction in root length of carrot and tomato seeds, with an RGI below 0.8 and a GI below 60% in all treatments. The AVB, OCT, and OXB mixtures resulted in an RGI lower than 0.8 for both plants and a GI lower than 60% in most treatments. Therefore, the lower concentrations of the filters individually showed no apparent phytotoxicity in carrot and tomato roots. In comparison, the higher concentrations, individually and in mixtures, were phytotoxic to the roots of these plants.

**TABLE 1:** Phytotoxic potential of the organic sunscreens avobenzone (1 and 10 ng/L), octocrylene (10 and 100 μg/L), and oxybenzone (2 and 20 μg/L), alone and in binary combinations, on rustic varieties of *Daucus carota* L. and *Solanum lycopersicum* L., based on the parameters seed germination, Relative Growth Index, and Germination Index.

AVB			
*D. carota*			
TR	G (%)	RGI ± SD	GI ± SD (%)
Co	98	1	100
1 ng/L	94	0.94 ± 2.0	88.7 ± 1.5
10 ng/L	92	0.45 ± 1.9*	49.5 ± 1.9*
*S. lycopersicum*			
TR	G (%)	RGI ± SD	GI ± SD (%)
Co	98	1	100
1 ng/L	96	0.96 ± 2.3	94.4 ± 1.7
10 ng/L	96	0.46 ± 2.5*	45.5 ± 2.0*

OCT			
*D. carota*			
TR	G (%)	RGI ± SD	GI ± SD (%)
Co	98	1	100
10 μg/L	94	0.84 ± 2.0	90.9 ± 2.0
100 μg/L	94	0.49 ± 1.5*	48.4 ± 2.0*
*S. lycopersicum*			
TR	G (%)	RGI ± SD	GI ± SD (%)
Co	98	1	100
10 μg/L	98	0.98 ± 2.5	98.1 ± 1.7
100 μg/L	96	0.51 ± 2.0*	49.3 ± 1.7*

OXB			
*D. carota*			
TR	G (%)	RGI ± SD	GI ± SD (%)
Co	98	1	100
2 μg/L	92	0.94 ± 1.8	85.9 ± 2.0
20 μg/L	94	0.46 ± 1.0*	52.3 ± 1.9*
*S. lycopersicum*			
TR	G (%)	RGI ± SD	GI ± SD (%)
Co	98	1	100
2 μg/L	98	0.96 ± 2.0	96.4 ± 2.5
20 μg/L	98	0.58 ± 2.8*	59.3 ± 2.5*

AVB‐OCT			
*D. carota*			
TR	G (%)	RGI ± SD	GI ± SD (%)
Co	98	1	100
1 ng/L × 10 μg/L	94	0.60 ± 1.5* ^,^ **	62.3 ± 1.0* ^,^ **
10 ng/L × 100 μg/L	90	0.50 ± 1.9*	56.0 ± 1.5*
*S. lycopersicum*			
TR	G (%)	RGI ± SD	GI ± SD (%)
Co	98	1	100
1 ng/L × 10 μg/L	94	0.63 ± 1.5* ^,^ **	59.7 ± 2.0* ^,^ **
10 ng/L × 100 μg/L	92	0.66 ± 2.0*	61.6 ± 2.0*

AVB‐OXB			
*D. carota*			
TR	G (%)	RGI ± SD	GI ± SD (%)
Co	98	1	100
10 ng/L × 20 μg/L	96	0.66 ± 1.5* ^,^ **	64.1 ± 1.9* ^,^ **
10 ng/L × 100 μg/L	90	0.26 ± 1.0* ^,^ **	22.6 ± 1.9* ^,^ **
*S. lycopersicum*			
TR	G (%)	RGI ± SD	GI ± SD (%)
Co	98	1	100
1 ng/L × 2 μg/L	100	0.65 ± 3.5* ^,^ **	65.1 ± 2.0* ^,^ **
10 ng/L × 20 μg/L	94	0.52 ± 3.5*	50.3 ± 2.0*

OCT‐OXB			
*D. carota*			
TR	G (%)	RGI ± SD	GI ± SD (%)
Co	98	1	100
10 μg/L × 2 μg/L	92	0.59 ± 3.9* ^,^ **	54.5 ± 3.0* ^,^ **
100 μg/L × 20 μg/L	90	0.63 ± 3.5*	53.7 ± 3.9*
*S. lycopersicum*			
TR	G (%)	RGI ± SD	GI ± SD (%)
Co	98	1	100
10 μg/L × 2 μg/L	100	0.51 ± 1.9* ^,^ **	52.4 ± 2.5* ^,^ **
100 μg/L × 20 μg/L	98	0.57 ± 2.5*	57.2 ± 2.0*

Abbreviations: AVB, avobenzone; Co, Control; G, seed germination; GI, Germination Index; OCT, octocrylene; OXB, oxybenzone; RGI, Relative Growth Index; SD, Standard Deviation; TR, Treatment.

* Significant difference in relation to the control.

** Significant difference in the mixture compared to their respective individual sunscreens, according to Kruskal‐Wallis H followed by Dunn's post hoc test (*p* ≤ 0.05).

**FIGURE 2:**
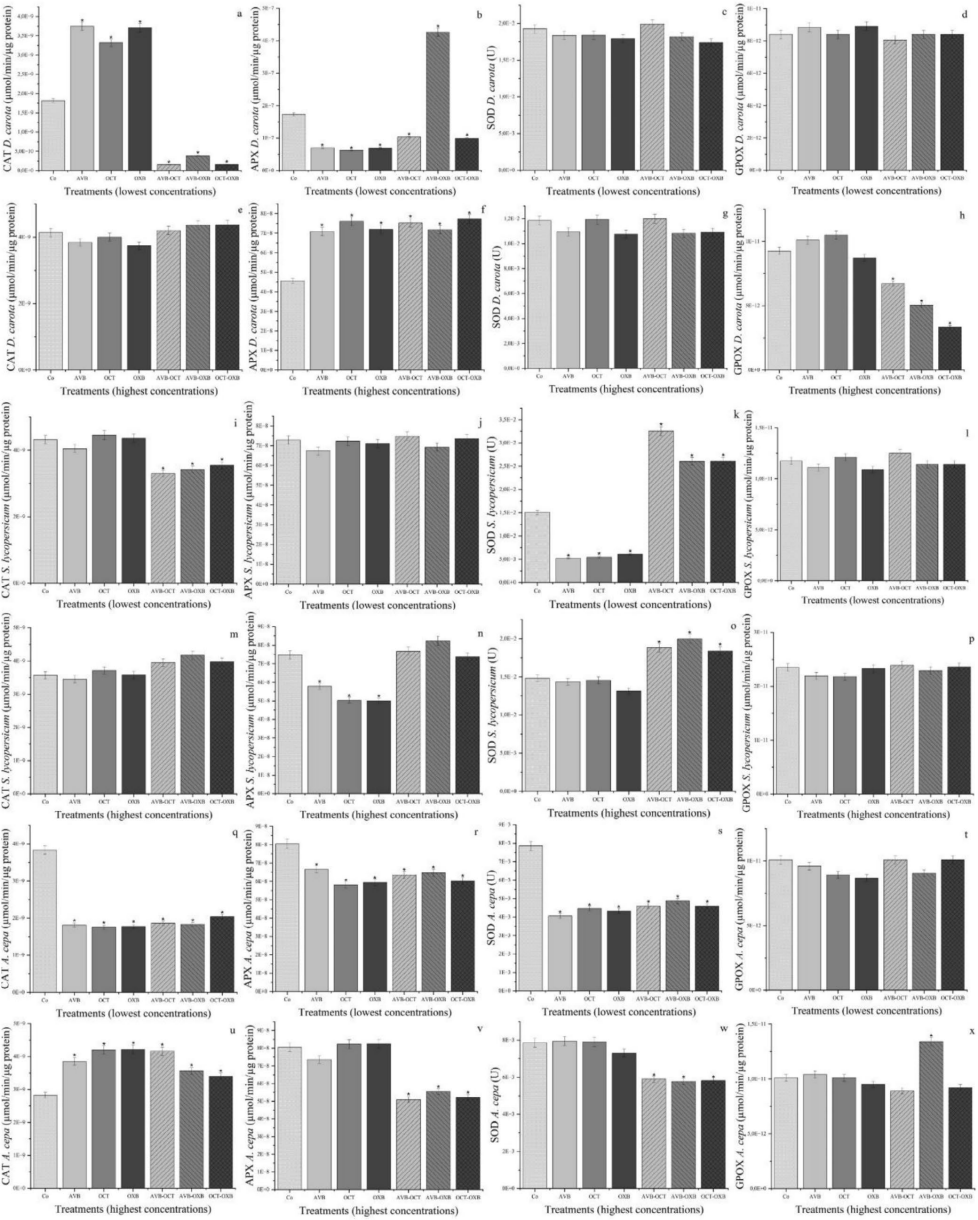
Modulations of the enzymes catalase (CAT), ascorbate peroxidase (APX), superoxide dismutase (SOD), and guaiacol peroxidase (GPOX) in seed roots of rustic varieties of *Daucus carota* L. and *Solanum lycopersicum* L., and in *Allium cepa* L. bulb roots exposed to the filters avobenzone (AVO, 1 and 10 ng/L), octocrylene (OCT, 10 and 100 μg/L), and oxybenzone (OXB, 2 and 20 μg/L). *Significant difference in relation to the control according to Kruskal‐Wallis H followed by Dunn's post hoc test (*p* ≤ 0.05).

The antioxidant enzymes CAT, APX, SOD, and GPOX (Figure 2) were analyzed in the roots of carrot and tomato seeds exposed to the two concentrations of filters alone and in mixture. These enzymes in plants maintain the homeostasis of cell division in meristems [8, 51]. In carrot, AVB, OCT, and OXB alone caused a significant increase in CAT and a reduction in APX at the lowest concentrations (Figure 2a,b), increasing the concentration of hydrogen peroxide in the roots to the point of inhibiting the activation of the ascorbate‐glutathione (AsA‐GSH) cycle. In tomato, the lower concentrations of AVB, OCT, and OXB alone did not alter the modulation of CAT and APX (Figure 2i,j). However, they significantly reduced the concentration of SOD (Figure 2k), probably increasing the concentration of superoxide radicals in the roots and, consequently, hydroxyl radicals.

The highest concentrations of AVB, OCT, and OXB alone in carrot did not alter the modulation of CAT and SOD (Figure 2e,g). However, they significantly increased the expression of APX (Figure 2f). In tomato, at the highest concentrations alone, APX was significantly reduced (Figure 2n), while the modulation of CAT and SOD did not change (Figure 2m,o). Both cases demonstrate the harmfulness of isolated UV filters to root tissues by increasing hydrogen peroxide in the cells, either via superoxide radicals, since SOD was not active, and/or by other oxidative processes triggered in the cells by these micropollutants. AVB, OCT, and OXB alone in carrot and tomato did not cause any change in GPOX modulation in any of the treatments (Figure 2d,h,l,p).

Therefore, based on the results in Figure 2, it can be inferred that all concentrations of AVB, OCT, and OXB alone, in carrot and tomato, increased the concentration of oxidizing compounds in the roots, mainly hydroxyl radicals and superoxides. Such oxidative radicals induce significant perturbations in the cell cycle, such as alterations in the assembly/function of the mitotic spindle, leading to disturbances in the distribution and movement of chromosomes during mitosis. They also cause the denaturation of enzymes such as those responsible for DNA replication, slowing or inhibiting tissue growth in plants [52, 53]. Thus, the oxidative stress triggered (Figure 2) corroborates the phytotoxicity observed for the highest concentrations of the filters alone (Table 1), which caused a significant reduction in radicle growth in carrot and tomato seeds.

However, the enzymatic results for carrot and tomato at the lowest concentrations (Figure 2a–d,i–l), which showed significant oxidative stress in the cells, contradict the macroscopic results of no phytotoxicity of AVB, OCT, and OXB isolated in the roots of these vegetables, as shown in Table 1. One explanation is that root growth is regulated by cell division in the meristems and cell elongation in the secondary tissues in the region proximal to the root tip. Thus, root development can be affected by inhibiting or interrupting one or both processes, mainly since they occur independently [54]. Thus, it is concluded that AVB, OCT, and OXB, isolated and at the lowest concentrations in carrot and tomato, although toxic to cell proliferation (Figure 2), did not affect the elongation of pre‐existing cells in the rootlets. According to Shishkova et al. (2008) [55], this condition ensures the functioning of the roots in the face of stressors, but only temporarily, since the number of cells does not increase and cell elongation has a limit, in addition to weakening them against friction.

According to scientific literature, the results obtained for root growth in rustic varieties of carrot and tomato for the highest concentrations of AVB, OCT, and OXB alone (Table 1) corroborate the conclusions of Beijora et al. (2024) [18], Nascimento et al. (2023) [8], Santo et al. (2023) [7], and Barros et al. (2023) [5], who observed root growth disturbances due to the accumulation of hydrogen peroxide in the cells of non‐rustic plant varieties exposed to these filters alone. However, in the studies by these authors, concentrations below 10 μg/L also reduced root growth, which differs from the results obtained here, where AVB, OCT, and OXB isolated in *D. carota* and *S. lycopersicum* at the lowest concentrations had root growth similar to their respective controls (Table 1), since cell elongation in the roots was not affected. This condition demonstrates a maneuvering mechanism, albeit provisional, of rustic varieties against these environmental contaminants.

In plants, CAT inhibition in the face of significant APX expression activates the AsA‐GSH cycle to maintain cell division and tissue development, increasing cellular reducing activity and endogenous antioxidant capacity to contain the production of oxidizing radicals [56]. This condition was observed for mixtures of AVB, OCT, and OXB filters in carrot at the lowest concentrations, in which CAT was strongly inhibited in AVB‐OCT, AVB‐OXB, and OCT‐OXB (Figure 2a), and APX was significantly activated in AVB‐OXB but inhibited in AVB‐OCT and OCT‐OXB (Figure 2b). Therefore, they were insufficient despite cellular efforts to preserve tissue function against AVB‐OXB by activating cell defense mechanisms such as the AsA‐GSH cycle. In roots exposed to AVB‐OCT and OCT‐OXB, there was no chance for the AsA‐GSH cycle to be activated at a level that would provide a protective benefit. In tomato, sunscreen mixtures at the lowest concentrations caused a significant decrease in CAT and a significant increase in SOD (Figure 2i,k). In contrast, at the highest concentrations, there was a slight increase in CAT and a significant increase in SOD (Figure 2m,o). In summary, for the mixtures, the three micropollutants, when combined, significantly increased the concentration of hydroxyl radicals and superoxide in the cells (Figure 2), causing a significant reduction in the growth of carrot and tomato seed radicles (Table 1).

In addition, the AVB, OCT, and OXB mixtures in carrot, at the highest concentrations, caused a significant reduction in GPOX (Figure 2h). The inactivation or significant reduction of this enzyme in plants triggers the oxidative degradation of lipids, generating free radicals in cells, mainly hydroxyl and hydroperoxyl radicals, which are very dangerous for meristems [51].

Based on the root growth results (Table 1), it can be inferred that AVB, OCT, and OXB in a mixture at lower concentrations had a synergistic interaction with each other, since the isolated filters had higher RGI and GI values than their respective mixtures. For the higher concentrations, it can be inferred that the combined sunscreens had an additive effect, since the RGI and GI values for them alone were similar to those obtained for their mixtures (Table 1). However, there was an exception to this condition at the highest concentrations: the AVB‐OXB combination had significantly lower RGI and GI than their respective filters alone (Table 1), demonstrating synergy between these filters.

### 3.3 Toxicity and Oxidative Stress in Roots of *A. cepa* Bulbs

Table 2 shows that all treatments significantly reduced cell division in *A. cepa* bulb roots, demonstrating a high mitodepressive effect and characterizing AVB, OCT, and OXB, alone and in mixtures, as cytotoxic. The MI observed for the mixed filter was less than 50% compared to the control, characterizes sublethal effect of the mixtures on root meristems.

**TABLE 2:** Analysis of the phytotoxic, cytotoxic, and genotoxic potential of the sunscreens avobenzone (1 and 10 ng/L), octocrylene (10 and 100 μg/L), and avobenzone (2 and 20 μg/L), alone and in binary combinations, based on the parameters Mitotic Index and Cell Alteration Index, in *A. cepa* bulb roots.

	TR	MI ± SD (%)	CAI ± SD (%)
Sunscreens	Co	100	0.4 ± 0.9
AVB	1 ng/L	63.7 ± 1.0*	3.4 ± 1.8*
	10 ng/L	62.7 ± 1.9*	7.0 ± 1.8*
OCT	10 μg/L	63.4 ± 1.7*	4.9 ± 1.2*
	100 μg/L	65.3 ± 1.0*	3.9 ± 1.9*
OXB	2 μg/L	63.3 ± 1.7*	2.9 ± 1.0*
	20 μg/L	53.4 ± 1.0*	8.2 ± 1.0*
AVB‐OCT	1 ng/L × 10 μg/L	45.1 ± 1.3*	20.0 ± 1.4* ^,^ **
	10 ng/L × 100 μg/L	43.7 ± 1.3*	22.4 ± 1.2* ^,^ **
AVB‐OXB	1 ng/L × 2 μg/L	40.3 ± 1.0*	22.5 ± 1.6* ^,^ **
	10 ng/L × 20 μg/L	35.1 ± 1.1*	31.7 ± 1.0* ^,^ **
OCT‐OXB	10 μg/L × 2 μg/L	48.1 ± 1.5*	25.9 ± 1.2* ^,^ **
	100 μg/L × 20 μg/L	44.5 ± 1.3*	33.9 ± 1.8* ^,^ **

*Note:* For MI, data is expressed as a percentage of Co values.

Abbreviations: AVB, avobenzone; CAI, Cell Alteration Index; Co, Control; MI, Mitotic Index; OCT, octocrylene; OXB, oxybenzone; SD, Standard Deviation; TR, Treatment.

* Significant difference in relation to the control.

** Significant difference of the mixture in relation to their respective sunscreens alone, according to Kruskal‐Wallis H followed by Dunn's post hoc test (*p* ≤ 0.05).

**FIGURE 3:**
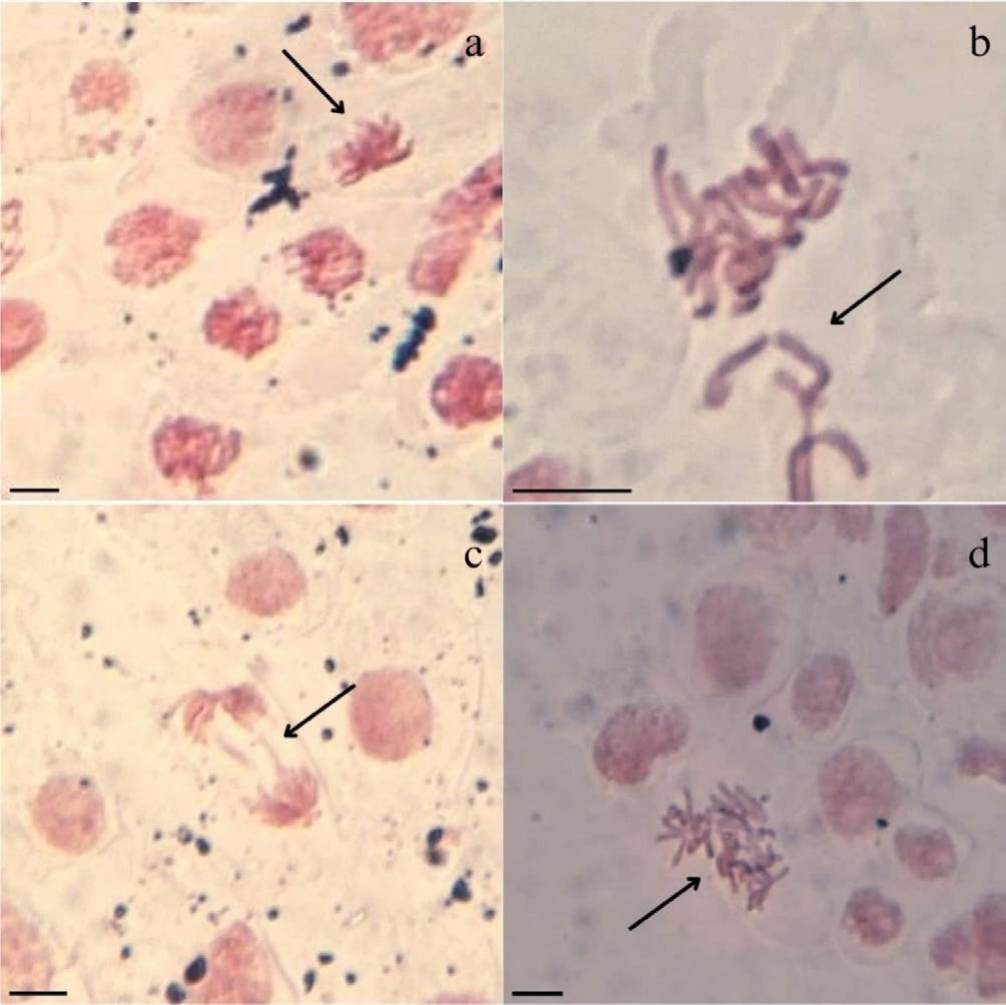
Cell alterations in root meristems of *Allium cepa* L. bulbs exposed to avobenzone (1 and 10 ng/L), octocrylene (10 and 100 μg/L), and oxybenzone (2 and 20 μg/L) in mixture: A) viscous metaphase; and individually and in mixture: B) disorder and chromosome loss in metaphase, c) bridge in anaphase, and d) chromosome disorder in anaphase. Bar: 10 μm.

Furthermore, Table 2 shows that AVB, OCT, and OXB, alone and in mixture, caused cellular changes in the root tips of *A. cepa*, revealing themselves to be genotoxic. In the mixtures, it can be observed that the rates of cellular changes increased significantly compared to the isolated filters, exceeding 20% in all treatments and reaching 31.7% for AVB‐OXB and 33.9% for OCT‐OXB (Table 2). The cellular alterations observed for the isolated and mixed treatments (Figure 3) were those resulting from disturbances in the assembly and/or function of the mitotic spindle, such as chromosomal disorder and chromosomal loss in metaphase (Figure 3b), bridging in anaphase (Figure 3c), and chromosomal disorder in anaphase (Figure 3d), classifying AVB, OCT, and OXB alone and in mixture as aneugenic.

In addition to the cellular changes mentioned above, binary combinations of AVB, OCT, and OXB at the highest concentrations induced metaphase viscosity (Figure 3a), increasing the aneugenic potential of the mixtures. It should be emphasized that viscosity is an alteration of the mitotic spindle due to the partial inactivation of the proteins responsible for DNA compaction. It is highly toxic, irreversible, and lethal [57, 58]. Thus, the mixtures' results of the intensification of cytotoxicity and genotoxicity in *A. cepa* (Table 2) demonstrated synergy between AVB, OCT, and OXB.

**FIGURE 4:**
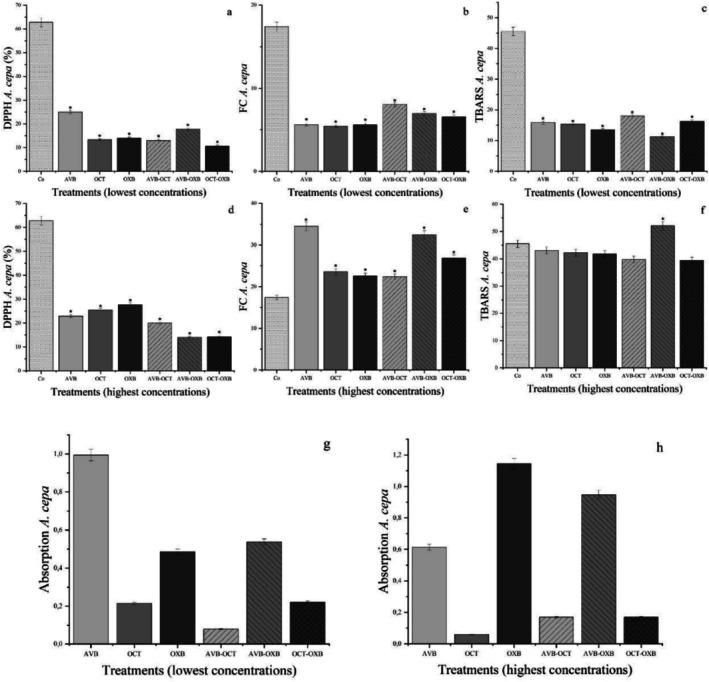
Modulations of antioxidant activity (DPPH), phenolic content (FC), and lipid peroxidation (TBARs) in bulb roots of *Allium cepa* L. exposed to the filters avobenzone (AVO, 1 and 10 ng/L), octocrylene (OCT, 10 and 100 μg/L), and oxybenzone (OXB, 2 and 20 μg/L), and absorption of the sunscreens, alone and in a mixture, by *Allium cepa* L. bulb roots. *Significant difference in relation to the control according to Kruskal‐Wallis H followed by Dunn's post hoc test (*p* ≤ 0.05).

Based on the non‐enzymatic biochemical analyses of *A. cepa* bulb roots (Figure 4), the lowest concentrations mixtures of AVB, OCT, and OXB reduced the concentration of antioxidant compounds (Figure 4a), consistent with the reduction of phenolic compounds (Figure 4b). At the highest concentrations, the filters alone and in mixtures, despite the significant reduction in the concentration of antioxidant compounds in general (Figure 4d), caused a significant increase in phenolic compounds, suggesting a cellular reaction against oxidizing radicals, but without protective effect (Figure 4e).

In addition, the enzymatic results in *A. cepa* roots, at the lowest concentrations, showed that the AVB, OCT, and OXB filters, individually and in mixture, caused a drastic inhibition of CAT, APX, and SOD (Figure 2q–s), corroborating the results of reduction in antioxidant compounds (Figure 4a) and phenolic content (Figure 4b) in the cells. These events increased the concentration of superoxide and hydroxyl radicals in the cells, causing reduction in cell division and significant number of cellular alterations in the roots (Table 2). Also, at the highest concentrations, AVB, OCT, and OXB filters, alone and in mixture, caused a significant increase in CAT (Figure 2u) and, in mixture, a significant reduction in APX and SOD (Figure 2v,w), increasing the concentration of these radicals in the cells and triggering significant cytotoxicity and genotoxicity in the root meristems. Regarding GPOX, combined with TBAR results (Figures 2 and 4), it can be seen that only AVB‐OXB, at the highest concentrations, induced lipid peroxidation in the roots (Figures 2x and 4f).

Therefore, the results obtained on *A. cepa* bulbs showed that AVB, OCT, and OXB, alone and in mixtures, at the concentrations evaluated, disarmed the plant defense system (Figures 2 and 4), increased the concentration of hydrogen peroxide in the cells, and induced significant cytotoxicity and genotoxicity in the root meristems (Table 2). However, the mixtures were more toxic than the filters alone because they increased the aneugenic potential in the roots.

The excess production of hydroxyl radicals and superoxides caused by AVB, OCT, and OXB, alone and in mixture, in seed roots and bulb roots (Figures 2 and 4) corroborates the studies by Zhong et al. (2020) [2], who irrigated seedlings of cucumber plants *Cucumis sativus* L. with different concentrations of OCT on the mg/L scale and found a reduction in the photosynthetic rate due to an increase in hydrogen peroxide in the leaves; Santo et al. (2023) [7] observed that OCT at μg/L caused sublethal effects in earthworms *Eisenia fetida* Sav. by increasing the concentration of superoxide and hydroxyl radicals in their tissues; Bordalo et al. (2022) [59] verified that AVB at μg/L increased the production of hydroxyl and superoxide radicals in the mussel *Mytilus galloprovincialis* L., inducing significant genotoxicity in these animals, and Gautam et al. (2022) [60] who exposed *E. fetida* earthworms to OXB in a subchronic treatment and found high levels of hydrogen peroxide in the cells of these organisms.

Therefore, the reduction in cell division and a significant increase in cellular changes (Table 2), together with the non‐enzymatic results (Figure 4) and those of antioxidant enzyme modulation (Figure 2) in the roots of *A. cepa* bulbs, corroborate and complement the toxicological results of AVB, OCT, and OXB, alone and in mixtures, on root growth in *D. carota* and *S. lycopersicum* (Table 1 and Figure 2). It can be inferred that the increase in superoxide and hydroxyl radicals in the rootlets of the seeds and the roots of the bulbs caused changes in the mitotic spindle, causing a significant mitodepressive effect in the meristems and, consequently, a reduction in root growth (Table 2). The results obtained for *D. carota*, *S. lycopersicum*, and *A. cepa* indicate that AVB, OCT, and OXB, alone and in mixtures, acted directly on the genetic material of the meristematic cells of the roots.

According to Gill et al. (2011) [61] and Sharma et al. (2017) [62], systemic and cellular toxicity resulting from oxidative stress—such as that induced in plants by mixtures of AVB, OCT, and OXB in the present study (Tables 1 and 2, Figures 2 and 4) – are among the significant causes of productivity loss in various crops worldwide by directly or indirectly affecting cellular, physiological, and morphological functions in plants. In addition to the yield factor, leaching from soils contaminated with these sunscreens threatens the survival.

Most mixtures evaluated here showed synergy between AVB, OCT, and OXB (Tables 1 and 2, Figures 2 and 4). Based on studies found in the literature on the ecotoxicological effects of organic sunscreens in mixtures, most of them showed a reduced or antagonistic toxic effect, as observed in Park et al. (2017) [25], who studied tertiary mixtures of ethylhexyl methoxycinnamate, OCT, and AVB in *Daphnia magna* L. and found a significant reduction in microcrustacean immobilization compared to the effect of the filters individually; in Du et al. (2017) [24], who studied binary mixtures of OXB and benzophenone‐4 in *D. magna*, *Brachydanio rerio* H., and *Chlorella vulgaris* B. and observed a significant reduction in mortality compared to the effects of these compounds alone; and González et al. (2022) [26], who found a significantly reduced toxic potential of ethylhexyl methoxycinnamate and OCT in *Paracentrotus lividus* L., *Acartia tonsa* D., and *Tisochrysis lutea* B. The fact that the toxicity of organic sunscreen mixtures is less harmful than individual filters to microalgae, microcrustaceans, copepods, sea urchins, and fish highlights the harmfulness of AVB, OCT, and OXB mixtures observed in plants (Tables 1 and 2).

The present study also evaluated the absorption capacity of onion roots to absorb the mixed filters. Figure 4 shows that AVB and OXB alone and the AVB‐OXB mixture were the treatments most absorbed by the roots of *A. cepa* (Figure 4g,h), demonstrating that the plants more easily assimilated the filters with lower hydrophobicity. The higher absorption efficiency of AVB‐OXB, compared to the other mixtures, explains the high phytotoxic, cytotoxic, and genotoxic effect of this mixture at higher concentrations in carrot, tomato, and onion roots, since it had significantly lower RGI and GI than AVB and OXB alone (Table 1), MI below 30%, high CAI (Table 2), and induced lipid peroxidation, in addition to showing a synergistic interaction in the three vegetables (Figures 2x and 4f). OCT, when present, caused a decrease in the absorption potential of the mixtures by the roots. However, the danger of this compound in combination with AVB and OXB stands out, because although they were the least absorbed mixtures, they were significantly harmful to plants and have high environmental stability.

## 4 Conclusions

AVB, OCT, and OXB, alone and in mixture, caused significant oxidative stress in the roots of *D. carota*, *S. lycopersicum*, and *A. cepa*, mainly by increasing the concentration of hydroxyl radicals and superoxides in the cells.

The lowest concentrations of AVB, OCT, and OXB alone, although toxic to the roots of *D. carota* and *S. lycopersicum*, did not cause a reduction in root elongation. However, they were very fragile.

The highest concentrations of AVB, OCT, and OXB individually, and all concentrations in mixture, were phytotoxic to the roots of *D. carota* and *S. lycopersicum*, and cytotoxic and genotoxic to the roots of *A. cepa*.

The AVB, OCT, and OXB mixtures demonstrated high toxicity to the evaluated plants, as they had a synergistic interaction with each other, being significantly more phytotoxic, cytotoxic, and genotoxic than the filters alone.

Using sewage sludge as fertilizer and wastewater in cropland irrigation is highly detrimental to agricultural productivity.

## Author Contributions

All authors contributed to the study conception and design. Material preparation, data collection, and analysis were performed by Diego Espirito Santo, Edson Araújo de Almeida, Regiane da Silva Gonzalez, Osvaldo Valarini Junior, and Ana Paula Peron. The final draft of the manuscript was written by Ana Paula Peron, with critical review for important intellectual content by Elisângela Dusman and A.C. Downs. All authors read and approved the final manuscript.

## Conflicts of Interest

The authors declare no conflicts of interest.

## Data Availability

Data sharing not applicable to this article as no datasets were generated or analysed during the current study.
